# Protecting noncommunicable disease prevention policy in trade and investment agreements

**DOI:** 10.2471/BLT.21.287395

**Published:** 2022-02-28

**Authors:** Anne Marie Thow, Amandine Garde, L Alan Winters, Ellen Johnson, Andi Mabhala, Paul Kingston, Pepita Barlow

**Affiliations:** a Menzies Centre for Health Policy and Economics, Level 2, Charles Perkins Centre (D17), University of Sydney, Sydney, NSW 2006, Australia.; b School of Law and Social Justice, University of Liverpool, Liverpool, England.; c Department of Economics, University of Sussex, Sussex, England.; d Faculty of Health and Social Care, University of Chester, Chester, England.; e Department of Health Policy, London School of Economics and Political Science, London, England.

## Abstract

Preventing noncommunicable diseases is a global priority, for which the World Health Organization has recommended policies to reduce the consumption of tobacco products, alcohol and unhealthy foods. However, regulation has been strongly opposed by affected industries, who have invoked the provisions of legally binding trade and investment agreements. The aim of this analysis of the legal, economic and public health literature was to present a short primer on the relationship between noncommunicable disease prevention policy and trade and investment agreements to help public health policy-makers safeguard public health policies. The analysis identified opportunities for protecting, and even promoting, public health in trade and investment agreements, including: (i) ensuring exceptions for public health measures are included in agreements; (ii) committing to good regulatory practice that balances transparency and cooperation with the need for governments to limit the influence of vested interests; (iii) ensuring trade and investment agreement preambles acknowledge the importance of public health; (iv) excluding investor–state dispute settlement mechanisms from agreements; and (v) limiting the scope and definition of key provisions on investor protection to reduce the risk of investment disputes. This synthesis of the multidisciplinary literature also provides support for greater strategic and informed engagement between the health and trade policy sectors. In addition, ensuring a high level of health protection in trade and investment agreements requires cooperation between disciplines, engagement with experts in law, economics and public health policy, and fully transparent policy processes and governance structures.

## Résumé

La prévention des maladies non transmissibles constitue une priorité mondiale. Dans ce contexte, l'Organisation mondiale de la Santé a recommandé des politiques visant à réduire la consommation de produits du tabac, d'alcool et d'aliments nocifs pour la santé. Néanmoins, cette régulation s'est heurtée à une forte opposition de la part des industries concernées, qui ont invoqué les dispositions prévues dans les accords juridiquement contraignants en matière de commerce et d'investissement. La présente analyse se penche sur la littérature juridique, économique et de santé publique. Son but: décrire brièvement la relation entre les mesures de prévention des maladies non transmissibles d'une part, et les accords en matière de commerce et d'investissement d'autre part, afin d'aider les législateurs compétents à défendre les politiques de santé publique. Cette analyse a identifié divers moyens de préserver, voire de promouvoir la santé publique dans les accords de commerce et d'investissement, notamment: (i) en veillant à inclure des exceptions pour les mesures de santé publique dans les accords; (ii) en s'engageant à respecter les bonnes pratiques réglementaires, qui concilient la transparence et la coopération avec la nécessité, pour les gouvernements, de limiter l'influence des intérêts en place; (iii) en s'assurant que les accords de commerce et d'investissement mentionnent dans leur préambule l'importance de la santé publique; (iv) en excluant de ces accords les mécanismes de règlement des différends entre États et investisseurs; et enfin, (v) en limitant la portée et la définition des dispositions fondamentales sur la protection des investisseurs afin de diminuer le risque de différend. Cette synthèse de littérature multidisciplinaire contribue également à plus d'engagement stratégique et éclairé entre les politiques sanitaires et commerciales. En outre, garantir un haut niveau de protection sanitaire dans les accords de commerce et d'investissement implique une collaboration entre plusieurs disciplines, la mobilisation d'experts en droit, en économie et en politiques de santé publique, ainsi que des structures de gouvernance et des processus d'élaboration des politiques entièrement transparents.

## Resumen

La prevención de las enfermedades no transmisibles es una prioridad mundial, por lo que la Organización Mundial de la Salud ha recomendado la adopción de políticas para reducir el consumo de productos de tabaco, alcohol y alimentos malsanos. Sin embargo, las industrias afectadas se han opuesto con firmeza a la regulación, invocando las disposiciones de los acuerdos comerciales y de inversión jurídicamente vinculantes. El objetivo de este análisis sobre la documentación jurídica, económica y de salud pública fue presentar una breve introducción sobre la relación entre la política de prevención de las enfermedades no transmisibles y los acuerdos comerciales y de inversión para ayudar a las autoridades responsables de formular las políticas de salud pública a protegerlas. El análisis identificó oportunidades para proteger, e incluso promover, la salud pública en los acuerdos comerciales y de inversión, que incluyen i) garantizar la inclusión de excepciones para las medidas de salud pública en los acuerdos; ii) comprometerse con las buenas prácticas regulatorias que equilibran la transparencia y la cooperación con la necesidad de los gobiernos de limitar la influencia de los intereses creados; iii) garantizar que los preámbulos de los acuerdos comerciales y de inversión reconocen la importancia de la salud pública; iv) excluir de los acuerdos los mecanismos de resolución de disputas entre inversores y Estados; y v) limitar el alcance y la definición de las disposiciones clave sobre la protección de los inversores para reducir el riesgo de disputas sobre las inversiones. Esta síntesis de la documentación multidisciplinar también respalda un mayor compromiso estratégico e informado entre los sectores de la salud y la política comercial. Además, se requiere la cooperación entre las disciplinas, la participación de especialistas en derecho, economía y política de salud pública, y procesos políticos y estructuras de gobernanza totalmente transparentes para garantizar un alto nivel de protección de la salud en los acuerdos comerciales y de inversión.

## ملخص

تعتبر الوقاية من الأمراض غير المعدية أولوية عالمية، وقد أوصت منظمة الصحة العالمية بسياسات للحد من استهلاك منتجات التبغ، والكحول، والأطعمة غير الصحية. إلا أن هذه القاعدة تعرضت للانتهاك الشديد من جانب الصناعات المتأثرة، والتي تحججت بأحكام اتفاقيات التجارة والاستثمار الملزمة قانونًا. كان الهدف من هذا التحليل للمؤلفات القانونية والاقتصادية، ومؤلفات الصحة العامة، هو تقديم كتاب تمهيدي قصير حول العلاقة بين سياسة الوقاية من المرض غير المعدي واتفاقيات التجارة والاستثمار، لمساعدة واضعي سياسات الصحة العامة في حماية سياسات الصحة العامة. حدد التحليل فرصًا لحماية الصحة العامة في اتفاقيات التجارة والاستثمار، بل والارتقاء بها، وتشمل هذه الفرص: (1) التحقق من إضافة استثناءات لتدابير الصحة العامة في الاتفاقيات؛ و(2) الالتزام بالممارسات التنظيمية الجيدة التي توازن بين الشفافية والتعاون من جهة، وحاجة الحكومات للحد من تأثير المصالح المكتسبة من جهة أخرى؛ و(3) ضمان إقرار ديباجات اتفاقيات التجارة والاستثمار بأهمية الصحة العامة؛ و(4) استبعاد آليات تسوية المنازعات بين المستثمرين والدول من الاتفاقيات؛ و(5) الحد من نطاق وتعريف الأحكام الرئيسية المتعلقة بحماية المستثمرين للحد من مخاطر منازعات الاستثمار. هذا التجميع من المؤلفات متعددة التخصصات يوفر كذلك دعمًا لمزيد من المشاركة الاستراتيجية والمستنيرة بين قطاعي السياسة الصحية والتجارية. بالإضافة إلى ذلك، فإن ضمان مستوى مرتفع من الحماية الصحية في اتفاقيات التجارة والاستثمار، يتطلب التعاون بين التخصصات، والمشاركة مع خبراء في القانون والاقتصاد وسياسة الصحة العامة، وعمليات سياسات وهياكل حوكمة شفافة بالكامل.

## 摘要

预防非传染性疾病是全世界的首要任务，世卫组织已建议实施政策以减少烟草制品、酒精和不健康食品的消费。但是，受影响的行业强烈反对该法规，他们援引了具有法律约束力的贸易和投资协议中的条款。对法律、经济和公共卫生文献进行分析是为了简要介绍非传染性疾病预防政策与贸易和投资协议之间的关系，以帮助公共卫生政策制定者维护公共卫生政策。该分析确定了在贸易和投资协议中保护、甚至促进公共卫生的机会，包括：(I) 确保协议中包含公共卫生措施的例外情况；(ii) 致力于良好监管实践，以平衡与政府的合作需求和透明度来限制影响既得利益；(iii) 确保贸易和投资协议序言承认公共卫生的重要性；(iv) 将投资者与国家之间的争端解决机制排除在协议之外；以及 (v) 限制主要条款对投资者保护的范围和定义，以降低投资纠纷的风险。综合多学科文献还为卫生和贸易政策部门之间制定更广泛的战略和知情参与提供了支持。此外，在贸易和投资协议中确保高水平的卫生保护需要法律、经济和公共卫生政策专家的参与和跨学科合作，以及完全透明的政策流程和治理结构。

## Резюме

Профилактика неинфекционных заболеваний является глобальным приоритетом, для обеспечения которого Всемирная организация здравоохранения рекомендовала применять политики по сокращению потребления табачных изделий, алкоголя и нездоровой пищи. Однако затронутые отрасли решительно выступили против регулирования, ссылаясь на положения юридически обязывающих торговых и инвестиционных соглашений. Цель данного анализа юридической, экономической литературы и литературы по общественному здравоохранению заключалась в том, чтобы представить краткое руководство по взаимосвязи между стратегией по профилактике неинфекционных заболеваний и торговыми и инвестиционными соглашениями с целью помочь ответственным лицам защищать политику в сфере общественного здравоохранения. В ходе анализа были выявлены возможности для защиты и укрепления здоровья населения в торговых и инвестиционных соглашениях, включая: (i) обеспечение включения в соглашения исключений для мер в сфере здравоохранения; (ii) приверженность надлежащей практике регулирования, которая уравновешивает прозрачность и сотрудничество с необходимостью для правительств ограничивать влияние корыстных интересов; (iii) обеспечение признания важности общественного здравоохранения в преамбулах торговых и инвестиционных соглашений; (iv) исключение из соглашений механизмов урегулирования споров между инвесторами и государством; (v) ограничение сферы охвата и определение ключевых положений о защите инвесторов для снижения риска возникновения инвестиционных споров. Обобщение междисциплинарной литературы также обеспечивает поддержку более стратегического и информированного взаимодействия между секторами здравоохранения и торговой политики. Кроме того, обеспечение высокого уровня охраны здоровья в торговых и инвестиционных соглашениях требует взаимодействия между дисциплинами, привлечения экспертов в области права, экономики и политики в сфере здравоохранения, а также полной прозрачности политических процессов и руководящих структур.

## Introduction

The prevention of noncommunicable diseases is a global priority given that they account for three quarters of deaths worldwide (i.e. 42 million annually).^1^ The direct economic costs of diabetes alone were estimated to be 760 billion United States dollars (US$) in 2019 and it is predicted that the cumulative loss to the global economy due to all noncommunicable diseases could exceed US$ 47 trillion by 2025.^2^^,^^3^ As the personal, economic and social burden continues to rise, there is a growing consensus that effective prevention of noncommunicable disease requires regulation of the tobacco, alcohol and food industries to reduce associated risk factors. In 2017 the World Health Organization (WHO) recommended a range of “best buy” policies to prevent noncommunicable disease, which included taxation, marketing restrictions and labelling of harmful products.^4^ Full implementation of these policies globally, particularly in low- and middle-income countries, could result in health-care savings and productivity gains valued at up to US$ 230 billion annually by 2030.^5^

Although governments have committed to regulating the tobacco, alcohol and food industries, imposing marketing restrictions, strengthening labelling requirements and introducing pricing policies,^6^ this regulation has been strongly opposed by industry.^7^^–^^9^ One strategy deployed to avoid or delay regulation has been to invoke legally-binding trade and investment agreements.^10^^,^^11^ Recent high-profile disputes have drawn attention to the potential for these agreements to constrain governments’ efforts to prevent noncommunicable disease. For example, the tobacco company Philip Morris challenged innovative tobacco packaging legislation by the governments of Australia and Uruguay.^7^^,^^10^^,^^12^^,^^13^ As a result of these disputes, public health actors are increasingly aware that trade and investment agreements can present barriers to robust prevention measures by constraining the policy space (i.e. the “freedom, scope, and mechanisms that governments have to choose, design, and implement public policies to fulfil their aims”).^14^

Public health and trade objectives are not mutually exclusive but they are often (and oversimplistically) perceived as such. Typically, trade and investment agreements contain clauses clarifying that nothing in the agreement prevents parties from addressing public health and human rights concerns.^9^^,^^15^ However, the scope and content of these clauses, and thus the protection afforded to health policy, vary. The public health community can help improve trade and health policy-making by engaging with negotiations on new trade and investment agreements and by highlighting how these agreements can protect, or even enhance, the policy space for preventing noncommunicable disease. Globally, changes in the (increasingly intertwined) trade and investment policy space have provided opportunities for public health engagement.^16^ In particular, many countries are negotiating or renegotiating trade and investment agreements, including the United Kingdom of Great Britain and Northern Ireland after its departure from the European Union and India and Brazil following the termination of investment treaties.^17^

Public health advocates and policy-makers often lament the lack of accessible information on opportunities for health protection in trade policy. Relevant information tends to be available primarily through specialist academic journals spanning multiple disciplines and may not be readily accessible to health policy-makers. In particular, trade and investment agreements, which are international legal agreements designed (primarily) to achieve economic policy objectives, are mainly subject to legal, policy and economic analyses. 

The aims of this article are to present a short primer on the relationship between noncommunicable disease prevention policy and trade and investment agreements and to identify mechanisms that can help safeguard public health policies. We draw on the legal, economic and public health literature, first, to describe the ways in which trade and investment agreements may constrain governments’ ability to enact best-practice, noncommunicable disease prevention policy and, second, to identify ways in which health policy actors can influence these agreements to optimize prevention policy.

## Trade and investment agreements

Trade and investment agreements encompass trade agreements, investment agreements, and combined trade and investment agreements and are generally based on a core set of provisions and principles. Trade agreements can be: (i) multilateral, which involve (nearly) all parties, such as World Trade Organization (WTO) agreements; (ii) plurilateral, which involve many parties; (iii) bilateral between two parties; or (iv) regional, with membership limited to a specified region. Investment agreements are mainly bilateral and combined trade and investment agreements are mainly bilateral or regional. Agreements are negotiated between countries (i.e. the parties), signed, implemented, administered and then enforced through agreed dispute settlement procedures, which include binding arbitration.

National governments participate in trade and investment agreements primarily to achieve economic objectives, such as: (i) reducing barriers to trade and investment flows; and (ii) creating a predictable regulatory environment for trade and investment. By signing an agreement, a government commits to ensuring their domestic policy measures are consistent with specific provisions of the agreement, which may be determined by binding dispute settlement mechanisms. However, trade and investment agreements also recognize necessary restrictions on trade, including policies implemented for public health purposes.^18^ Non-discriminatory public health measures that are designed to achieve legitimate objectives without imposing unnecessary restrictions on trade are usually permissible. The key terminology is explained in Box 1.

Box 1 **Trade and investment agreement terminology relevant to noncommunicable disease prevention**

**Dispute settlement mechanism:** a formal structured process that addresses disputes between parties to a trade and investment agreement. Disputes can also be raised by investors against parties (i.e. national governments) under investor–state dispute settlement mechanisms. Dispute settlement is mediated by courts, tribunals or other adjudicatory bodies and decisions are binding on parties to the agreement.

**Exception:** exceptions to commitments contained in trade and investment agreements are permitted and may be based on the recognition that trade and investment operate in a context in which governments also pursue other public interest objectives. Exceptions must be legitimate and necessary.

**Expropriation:** may occur when property is taken by the state for public use or benefit. Indirect expropriation can occur if new regulations undermine the ability of an investor to take full advantage of their investments.

**Fair and equitable treatment:** the concept is frequently invoked in investment disputes and refers to the standard set by international law for the way states must treat the property of foreign nationals. Fair and equitable treatment is determined by reference to specific circumstances and not, for example, by treatment accorded to other investors.

**Increasing economic efficiency:** improving the allocation of resources to increase economic welfare. In the context of trade and investment agreements, this may involve minimizing the diversion of trade, the impact of regulatory diversity on international trade and other market distortions.

**Legitimacy:** whether a domestic measure pursues legitimate public interest objectives and can contribute effectively to these objectives. Arbitrary discrimination and disguised restrictions on trade are not legitimate public interest objectives.

**Legitimate expectations:** form part of the standard for fair and equitable treatment and relate to due process. The law must be applied consistently in light of the representations (e.g. commitments related to future policy measures) made by a host state, thereby enabling individual investors to rely on those representations. The extent to which legitimate expectations have been established influences the application of exceptions and the interpretation of investors’ rights.

**Necessity (proportionality):** requires consideration of the extent to which a measure unduly restricts trade or investment and, in particular, of whether an equally effective alternative measure could achieve the objective or objectives pursued while being less restrictive of trade or investment.

**Non-discrimination:** according to the World Trade Organization, non-discrimination involves two elements: (i) all parties (i.e. other countries) should be treated without discrimination (i.e. according to the most favoured nation principle; however, bilateral and regional trade and investment agreements are conditional violations of the most favoured nation principle); and (ii) all like goods, services and nationals (whether of domestic or international origin) should be treated equally once they have entered the country, paid any taxes required and met local standards (this is known as the principle of national treatment).

**Predictability:** the creation of certainty and confidence regarding the trade- and investment-related policy environment. For example, trade barriers or requirements for investors should not be introduced arbitrarily and investors’ rights in the host country should be protected.

## Constraints on prevention policy

Both industry and governments have invoked commitments in trade and investment agreements to delay or avoid the implementation of noncommunicable disease prevention measures. This approach has occurred most clearly in formal disputes involving dispute settlement mechanisms. However, informal challenges are more frequent; for example, when specific trade concerns affecting the implementation of noncommunicable disease prevention policies have been raised in WTO committees.^19^

Formal disputes and informal challenges can also contribute to wider changes in noncommunicable disease prevention policy or to the abandonment of a policy altogether (i.e. regulatory chill).^20^^,^^21^ Governments are sometimes reluctant to adopt novel measures when: (i) a dispute has occurred elsewhere; or (ii) there is the threat of a dispute. For example, the New Zealand government delayed the introduction of tobacco plain packaging legislation while awaiting the outcome of disputes about Australian regulation.^21^ This attitude is problematic because the threat of a dispute does not necessarily mean the challenge will be successful.^8^ In these circumstances, the tobacco, alcohol or food industry may feel emboldened to pressure governments to change course strategically, even when the chance of success in a dispute is small. Therefore, it is not surprising that, over the past 20 years, the number of investor–state disputes has increased while the success rate has decreased, perhaps as the “result of a rise in strategic litigation by investors whose aim is not only to obtain compensation but also to deter governments’ regulatory ambitions.”^22^

Table 1 summarizes the key commitments in trade and investment agreements that can be used to constrain public health policy on noncommunicable disease prevention, as identified in legal, economic and public health analyses. Two widely cited clauses are: (i) the commitment to provide compensation for indirect expropriation, which can occur when government policy detrimentally affects an investment; and (ii) investor–state dispute settlement mechanisms (Box 1). Other clauses involve commitments: (iii) to limit technical barriers to trade; (iv) to extend protections for intellectual property rights; (v) to ensure good regulatory practice; and (vi) to ensure fair and equitable treatment. Although these provisions are all designed to achieve the objectives of trade and investment agreements, the extent to which they can be used to challenge noncommunicable disease prevention policies could be influenced by paying attention to the health implications of agreements when they are being drafted.

**Table 1:** Commitments in trade and investment agreements used to constrain noncommunicable disease prevention policy

Trade and investment agreement commitment	Summary of relevant features	Examples of use to constrain noncommunicable disease prevention policy
**Limits to technical barriers to trade**	Limits the introduction of new regulations (including food, alcohol and tobacco regulations) to ensure they do not introduce unnecessary trade costs if, for example, the objective could be achieved through other means. These limits typically accompany rules requiring parties to an agreement to share information about new regulations and to provide opportunities for stakeholders to comment before their adoption.^19^ Demonstrating that a new regulation is necessary despite its impact on traded goods can involve onerous evidence requirements^23^^,^^24^	Can be invoked to challenge new regulations. For example, parties can argue that a new regulation should be weakened or that an alternative should be pursued
**Increased protections for intellectual property rights**	Commitments to protection of intellectual property rights, particularly trademarks^7^^,^^25^^,^^26^	Can be invoked to challenge noncommunicable disease policies that affect labelling, packaging or advertising, particularly policies that affect the use of trademarks
**Limits to indirect expropriation**	Limits to states’ ability to introduce new regulations that undermine the ability of the investor to take full advantage of their investment as anticipated^26^	Can be invoked to challenge noncommunicable disease policies that affect the value of an international business’s investments, including in some cases future anticipated profits
**Fair and equitable treatment commitments**	Protections against unfair treatment resulting from a regulation being introduced. Can include protections against the introduction of measures deemed arbitrary, discriminatory or unreasonable^26^^,^^27^	Can be invoked to argue that a new noncommunicable disease policy is discriminatory because, for example, it affects certain products associated with the investment but not others
**Good regulatory practice provisions**	Requirements affecting the development of new policies, including requirements on transparency and stakeholder consultation. Can also include commitments to allow stakeholders from all parties to an agreement to participate in policy development^28^^,^^29^	Provide new opportunities for industry actors with a conflict of interest to request participation in policy development processes and to lobby against the introduction of effective noncommunicable disease policies

## Drafting agreements

Analyses from the legal, public health and economic literature indicate that carefully drafting trade and investment agreements to explicitly articulate commitments to public health can help governments pursue public health objectives and create expectations among parties that health policy will be evidence-based,^23^^,^^30^ thereby reconciling states’ trade and public health obligations.^9^ In particular, careful drafting can provide explicit recognition that a state may need to impose trade barriers to protect and promote the human right to health.^10^^,^^31^ Here we discuss four mechanisms for achieving this outcome.

### (i) Exceptions for public health measures

General exceptions in trade and investment agreements typically state that nothing in the agreement should prevent a party from adopting measures necessary to protect public health (often expressed in a list of other legitimate public interest measures), provided they are non-discriminatory and do not constitute an unnecessary restriction on trade. Exceptions are thus important for providing policy space for noncommunicable disease prevention measures that affect traded goods or services or the products of investment.

The application of these exceptions is usually explicitly premised on compliance with the legal principles underpinning trade agreements, such as non-discrimination and necessity.^32^^,^^33^ For both trade agreements and integrated trade and investment agreements, establishing the necessity of a measure and whether it can benefit from an exception relies on a clear explanation of how the measure contributes to its objectives. Critical factors are evidence supporting the measure’s effectiveness and a determination that the measure does not unduly restrict trade (and that there are no viable alternatives that are less trade-distorting).^24^^,^^25^^,^^30^

### (ii) Limiting the risk of investor–state disputes

The wording of provisions on investor protection is important for safeguarding the policy space for noncommunicable disease prevention measures because these measures may affect investors in the tobacco, food or alcohol industries. Public health measures can be protected by excluding investor–state dispute settlement commitments from trade and investment agreements, as in the Australia–United States Free Trade Agreement.^34^ Another option is to carve out (i.e. exclude) specific measures from an investor–state dispute settlement; this was achieved for tobacco control measures in at least three recent agreements.^34^ Alternatively, indirect expropriation could be ruled out as a basis for an investor–state dispute settlement claim, as in the United States–Mexico–Canada agreement,^35^ or public health measures could be excepted from the expropriation chapter, as in the United States–Republic of Korea Free Trade Agreement.^24^ Provisions on fair and equitable treatment commonly provide a basis for investor–state dispute settlement claims and are broadly recognized as excessively ambiguous.^36^ Consequently, a clear definition of fair and equitable treatment, which includes qualifications to limit application of the concept, can help safeguard public health policies.^23^

Another approach is to ensure trade and investment agreements include a clear definition of indirect expropriation that limits the extent to which it applies to public health measures. For example, in the European Union–Canada Comprehensive Economic and Trade Agreement and the European Union–Singapore Trade Agreement, the definition of indirect expropriation includes the following: “The determination of whether a measure constitutes an indirect expropriation requires a case-by-case, fact-based inquiry… For greater certainty, except in… rare circumstances… a non-discriminatory measure or series of measures by a Party that are designed and applied to protect legitimate public objectives such as public health, safety and the environment, do not constitute indirect expropriation.”^27^ This clause effectively disallows claims of indirect expropriation for legitimate, non-discriminatory and necessary public health measures.

### (iii) Trade and investment agreement preambles

The inclusion of statements in preambles to trade and investment agreements that recognize the importance of public health as a whole-of-government objective creates the expectation that governments will implement (and prioritize) future public health measures. Consequently, preambles can aid the interpretation of contentious provisions, even though these statements are not legally binding.^23^^,^^30^ For example, the preamble to the 2018 Peru–Australia Free Trade Agreement recognizes the “right to regulate and… to set legislative and regulatory priorities… and protect legitimate public welfare objectives, such as public health….”^37^ In addition, governments can enhance the effectiveness of such statements in preambles by ensuring they do not lead investors to expect that the public health regulatory environment will remain unchanged.^38^

### (iv) Good regulatory practice provisions

Provisions on good regulatory practice that give governments the flexibility to determine appropriate stakeholder engagement, thereby minimizing conflicts of interest, are important for supporting evidence-based, best practice, public health policy. One option is to exclude the good regulatory practice chapter from dispute settlement mechanisms to avoid conflicts over differing interpretations, as was done in the Trans-Pacific Partnership regulatory coherence chapter.^28^ Alternatively, qualifications to good regulatory practice clauses that refer to “appropriate” consultation or engagement could help manage conflicts of interests and promote effective noncommunicable disease prevention policies.^39^

## Protecting health through agreements

The literature we reviewed indicates that the health sector can protect the policy space governments have for implementing noncommunicable disease prevention policy measures in the context of trade and investment agreements in two main ways: (i) through health policy design; and (ii) through engagement with the trade sector.

First, health policy measures for noncommunicable disease prevention can be strategically designed to minimize the risk of violating trade and investment agreement commitments; in effect, public health benefits are maximized by understanding the constraints presented by these agreements.^40^ In particular: (i) key trade and investment agreement principles, such as necessity and non-discrimination, should be taken into account when articulating the objectives and substance of a health measure; (ii) evidence for the necessity of the measure should be documented; and (iii) good regulatory practice provisions should allow actions to minimize conflicts of interest.^24^^,^^41^ Reference to international instruments, such as WHO’s Framework Convention on Tobacco Control, can also strengthen policy measures for noncommunicable disease prevention.^12^^,^^13^^,^^15^ The Framework Convention has been used repeatedly as a reference point by tribunals “who reliably uphold non-discriminatory [tobacco control measures] on public health grounds.”^42^ In addition, it has been specifically cited as providing supportive evidence for the necessity of tobacco policy measures in formal disputes and other challenges under trade and investment agreements.^13^

Health policy design is also particularly relevant for safeguarding noncommunicable disease prevention policy when considering commitments on technical barriers to trade contained in trade and investment agreements. Usually there is no specific health exception to commitments on technical barriers to trade that could be applied to, for example, noncommunicable disease prevention policy measures concerning diet or tobacco or alcohol consumption. Nevertheless, trade and investment agreements explicitly recognize health as a legitimate objective for which technical measures can be adopted; for example, see article 2.2 of the WTO Technical Barriers to Trade Agreement,^43^ which is often included in its entirety in new trade and investment agreements. The unsuccessful dispute about Australia’s tobacco plain packaging policy under the WTO Technical Barriers to Trade Agreement showed that recognizing the public health objective of the measure (as a legitimate objective) was critical.^12^ The central issues were the necessity of the measure and whether the objective could be achieved through another less trade-restrictive measure.^12^ For example, the main objective of a labelling policy measure could be its immediate effect of informing consumers rather than its long-term effect on health indicators, such as the prevalence of obesity or noncommunicable disease.^44^

Second, cross-government engagement between the health and trade sectors during negotiations can draw attention to potential constraints on health policy that may arise from trade and investment agreements. A core tenet of trade policy is the need for complementary and mitigating policies to address the unintended consequences of a trade and investment agreement and maximize its benefits; when an agreement places constraints on health policy, the health sector may be unable to implement mitigating policy measures. Often public health actors do not contribute to negotiations, either through lack of knowledge or interest or because they are deliberately excluded.^45^^,^^46^ However, the health sector can expand policy space for noncommunicable disease prevention during the drafting of trade and investment agreements given: (i) awareness of the key provisions that could be raised for consideration; (ii) familiarity with health protections in existing agreements; and (iii) access to legal and economic expertise on trade. At the national level, the health sector could participate in formal consultations, submissions and direct lobbying during the drafting and negotiation of agreements.^46^^,^^47^ For example, the use of formal and informal mechanisms to raise awareness of protecting access to medicines and tobacco control was important for including safeguards in Australia’s negotiating agenda for the Trans-Pacific Partnership.^47^

Experience to date indicates that opportunities for cross-sectoral engagement can be maximized by ensuring that trade and health policy-makers have the capacity to facilitate constructive participation by public health actors.^9^^,^^23^^,^^48^ For example, the strength of governmental and nongovernmental health sector actors contributed to the removal of proposed intellectual property and investment provisions in the Regional Comprehensive Economic Partnership in Asia and the Pacific region that could have limited access to medicines.^49^ The literature shows that trade negotiations need to provide formal opportunities for health sector engagement and should be more transparent. Increased engagement by the health sector could provide a clear understanding of the health sector’s requirements for specific inclusions in trade and investment agreements (to inform negotiations) and could ensure that the implications of a proposed agreement are thoroughly analysed.^46^ An important consideration is that industry can have a strong influence on the extent to which a trade and investment agreement is interpreted as placing constraints on noncommunicable disease prevention policy.^50^ Building capacity in the health sector could provide alternative interpretations of the need for a specific health policy measure that satisfies commitments within these agreements.^48^ In addition, engagement by global health policy actors can provide normative support for national health policy, as well as technical and evidential support for noncommunicable disease prevention policy measures.^13^^,^^26^^,^^48^

## Conclusion

Our analysis has identified opportunities for protecting, and even promoting, public health (particularly noncommunicable disease prevention policy) in trade and investment agreements by, for example: (i) ensuring exceptions for public health measures; (ii) excluding investor–state dispute settlement mechanisms; (iii) clearly defining fair and equitable treatment and indirect expropriation and limiting their potential to cause disputes; (iv) ensuring good regulatory practice provisions balance transparency and cooperation with the need to limit the influence of vested interests; and (v) ensuring trade and investment agreement preambles acknowledge the importance of public health.

We also found that a high level of health protection in trade and investment agreements requires cooperation between several disciplines. Trade and investment lawyers can help design health policy measures that regulate commercial activities and can anticipate and counter legal challenges under trade and investment agreements. Cross-sectoral engagement between trade and health policy-makers is also critical. Trade policy-makers should recognize the importance of health and engage with the health sector during trade negotiations. Health policy-makers should engage with experts in law, economics and public health policy to ensure negotiations are informed by feasible proposals that promote better public health. These strategies will be most effective when policy processes and governance structures are fully transparent.
